# A Clinical Lecture on Infantile Paralysis

**Published:** 1906-08-04

**Authors:** George Parker

**Affiliations:** Physician to the General Hospital, Bristol.


### Hospital Clinics.

### A CLINICAL LECTURE ON INFANTILE PARALYSIS.

By George Parker, M.A., M.D.Cantab., Physician to the General Hospital, Bristol.

One of the most common causes of deformities in the limbs is an attack of infantile paralysis, and while it is one which can neither be foreseen nor prevented, a great deal can be done to lessen its effects. I propose to show how the disease is to be diagnosed in the early stages, and what can be done to remedy the injuries it produces. Though it is very rarely fatal, the patient is left more or less crippled for life.

### Diagnosis.

In the primary stage it is not often seen; and, when seen, we are apt to mistake it for rheumatic fever, influenza, or an obscure pneumonia. The ordinary form is, like them, an acute infection, which occurs more frequently in the hot months, and has been occasionally met with in small epidemics. A comparison may be drawn between infantile paralysis and herpes zoster, outbreaks of which arise in a similar manner; but infantile paralysis more rarely attacks adults than herpes does, and the seat of the lesion is in the anterior horns of the cord producing motor paralysis and muscular atrophy, while herpes zoster is due to a lesion in the posterior horns, and is not followed by paralysis.

The subjects of an attack are usually about two or three years of age, though instances occur in much younger or older children, and rarely in adult life. In the majority of cases the onset is marked with fever, pains in the back and limbs, or a gastro-intestinal attack. Not infrequently this comes on when the patient is convalescent and weak after some other disease, such as typhoid or diphtheria. The child may cry from the pains about the joints, and in the course of two or three days one or more limbs are found to be paralysed, while the fever subsides in the course of a week. Another mode of onset occurs in some patients who, according to Starr, amount to about a quarter of the whole number. Here sudden paralysis appears without fever or pain. Sometimes it is noticed on their first waking in the morning, sometimes it follows a slight injury or shock, or in boys after prolonged bathing in cold water. Whatever be the mode of onset, the attack results in a uniform type of paralysis of peculiar distribution, with atrophy of the muscles, but no loss of sensation, no spasm or rigidity, and no mental affection. The phases through which the patient passes are (1) the febrile stage of about a week; (2) the stage of widely extended paralysis, lasting six or twelve weeks; (3) the stage of decreasing paralysis from the second to the twelfth month; and lastly (4) the condition of residual paralysis which is lifelong, though capable of much amelioration. It has been suggested that occasionally abortive attacks occur which recover completely in the early period, but it is doubtful whether this ever really happens. In practically all cases the paralysis is more extensive during the first few weeks than later on. The reason of this is said by some authorities to be that the disease produces inflammatory changes in the anterior horns of certain segments of the spinal cord. Here the central branches of the anterior spinal artery are dilated and surrounded by a zone of cellular infiltration. Some nerve cells are destroyed at once, and others are compressed by the tissue congestion, but as this passes away those which are merely compressed recover. The course of events certainly tallies with this theory, but the exact pathology is by no means certain. When the so-called inflammatory stage is over, the muscles supplied by the permanently damaged nerve-cells have begun to atrophy, the reaction of degeneration has commenced in them, and the tendon jerks which are dependent on them have ceased for a time, if not for ever. The other muscles, however, gradually recover their functions, and thus paralysis disappears over greater or smaller areas. In the limbs which remain affected we are struck with the coldness and blueness of the tissues, and, as time goes on, with the comparative loss of growth in the bones.

### Distribution of Injuries.

Before we proceed to discuss the diagnosis from other forms of paralysis in children, it will be well to consider the distribution of the injuries. The first point is the way in which individual muscles or groups are picked out apparently at random, but really according to the arrangement of the cells in the anterior horns which govern those muscles. Some of the groups of cells which supply a given muscle extend through several segments. Hence a lesion in one segment may entirely paralyse certain muscles and only weaken others. Thus, too, a group of muscles may be more or less affected and atrophied, though adjoining muscles innervated from another segment are unaffected. If serious damage has occurred in the fifth and sixth cervical segments we get paralysis of muscles about the shoulder, together with the biceps and supinator longus. If the seventh or eighth is affected such muscles as the long extensors or the long flexors of the fingers may be involved. Similarly an injury of the upper lumbar segments may paralyse some of the thigh muscles, while a sacral lesion affects certain muscles of the leg and foot. Perhaps the most common paralyses are seen in the shoulder muscles, or the extensors of the wrist, and as to the lower limb in the distal muscles in front of the leg—*i.e.* the anterior tibial and the peroneal groups—while the calf and hamstring muscles generally escape. Various combinations may occur, such as the shoulder and intrinsic muscles of the hand, or a group in one arm and the opposite leg.

### Secondary Deformities.

Now paralysis of such individual muscles or groups affects their fellow-workers and antagonists. The healthy unopposed muscles draw the limb into abnormal positions, the weakened groups are overbalanced and hyperextended, which weakens them still more, while the contracted but healthy muscles become permanently shortened. Thus, though the paralysis causes no spasm or rigidity directly, the antagonist muscles develop some degree of contraction indirectly. These deformities are increased by the effect on the joints of the loss of so many muscles which would normally support them. The head of the humerus, for instance, drops out of its socket, the foot is terribly distorted, talipes in various forms is set up, adhesions and bony changes follow, and the patient, if untreated, may suffer more from these secondary deformities than from the paralysis itself. It is astonishing how the atrophied muscles, if deformities are prevented, will rise to their work. I have seen a young fellow who won a hundred yards' race with a leg little thicker than a broom-stick. A few remaining healthy fibres will develop into a useful muscle if they are exercised and not dragged into a position of disadvantage. Hence the use of mechanical supports just so far and so long as they do not interfere with the action of the weakened muscles, while they prevent them being forced out of their position.

### Difficulties with Young Children.

The diagnosis in very young children may be difficult if electrical tests cannot be made, or the voluntary movements elicited. Of course rheumatic and tuberculous joint disease may occasion some immobility of a limb, though, by the way, rheumatism does not very often attack the joints in children. A rickety child, or one with hip-joint disease, might be mistaken for one in the early acute stage of infantile paralysis, where pain still exits. Thus a boy who has now one flaccid wasted leg began with pain in both knees, increased by any movement. After the febrile stage is over we may have to distinguish cases of infantile paralysis from (1) *Erb's or Duchesne's paralysis* due to a lesion of the fifth or sixth cervical nerves. Here there is a history of an injury or difficult birth, such as a breech presentation, and generally some anæsthesia exists in the area of the circumflex nerve. Again, if a muscle, such as the triceps, is implicated, which is not normally connected with those two nerves, then there is reason for deciding in favour of infantile paralysis and giving a less favourable prognosis.

A more common error is to mistake (2) a *cerebral paralysis.* Here there is little atrophy, though a whole side or limb may cease to grow, the tendon-reflexes are preserved or exaggerated, some rigidity or athetosis is present, and the Faradic reaction is perfect. The mental condition of these children, too, is often feeble and unstable. Convulsions may recur from time to time, and the spastic condition of the muscles requires very different treatment.

(3) *Birth Paralysis* or Little's disease, though it presents very variable symptoms as to the paralyses and mental state, always shows spastic inco-ordination. The tendon reflexes are exaggerated unless the rigidity has become structural and too great to permit them to take place. Individual muscles are wasted from disuse, their movements being prevented by the tonic spasm of other muscles. In addition to this there is a history of the trouble dating from birth and of injuries at that time.

(4) *Multiple neuritis* is accompanied with much pain, but is said to occasionally follow or to occur with infantile paralysis. The ordinary, form, however is bilateral and symmetrical, and the distribution of the paralyses differs from that due to lesions in spinal segments (5) *Myopathic muscular* atrophy such as hypertrophic paralysis may in rare instances give rise to some difficulty, but the electrical reactions are not altered, and there is no real paralysis. (6) *Diphtheritic paralysis* is a form of neuritis, and even if the history cannot be relied upon, the anæsthesia and the symmetrical distribution should enable us to recognise it. The distribution of the lesions again should help us to exclude (7) any form of myelitis or pachymeningitis which might otherwise resemble infantile paralysis.

### Treatment.

As to treatment, if the patient is seen in the acute stage, complete rest in bed with the usual care of a febrile attack should be ordered. Linseed poultices with mustard may be applied along the spine, and iodides and ergot may also be given. Later on the child must still be kept warm, general massage may be employed, and voluntary movements should be encouraged when the inflammatory condition has passed away. The electrical reactions should be tested, the greatest care being taken not to unduly alarm the patient. Those muscles in which the Faradic reaction remains will recover, those in which the reaction of degeneration appears will be more or less damaged. After the first fortnight, electrical changes can generally be made out, the galvanic reaction in the damaged muscles is increased for some months and may finally disappear altogether like the Faradic reaction. The latter, which disappears early, may sometimes return in muscles which recover. We should also test the voluntary movements which are left, by tempting the child to use the paralysed limb and noting the results. Having thus ascertained the extent of the paralysis, we give regular local massage for the affected muscles, and graduated exercises for the weak ones with steady electrical treatment. Next it must be considered what deformities are likely to result, and measures must be taken to prevent them, since the deformities and contraction of the uninjured muscles bear such an important part in the general result.

The massage and electricity are required to maintain the nutrition of the weakened muscles until the return of nerve action, and to increase their growth after the stage of amelioration has passed. In what way shall we best use electricity?

Now the Faradic current is painful for children, and some writers doubt its value for muscles which do not contract under it. The galvanic and sinusoidal currents are much less unpleasant, especially if they are given through pans of water in which the foot or arm are immersed, but in private houses the apparatus is difficult for the nurse to manage. When the alternating current is used for light, a simple and safe transformer can be made by interposing a lamp and a secondary coil from a Faradic battery with the hammer screwed down. Whatever means are used, the child should for some days have applications without any current, in order that he may become accustomed to it, and then only the mildest strength should follow. It is astonishing how in this way all excitement may be avoided. Afterwards the current—at a strength of 8 or 10 milliampères—should be continued daily for six or twelve months. Galvanic electrodes must never be left long on the same spot of skin for fear of burns, and any scratches should be covered with collodion if the child is to bear treatment quietly. Massage should be given continuously and is one of the most effective modes of treatment. We do not expect that electricity will affect the lesions in the cord, but it has some advantage in aiding the growth of the muscles, as Debedat showed by experiments in which one limb thus treated could be compared with another which was left alone.

Exercises should be devised, such as those in Swedish drill, squeezing a ball or the use of dumb-bells, and every effort should be made to get the child to use the weakened muscles constantly and vigorously.

Strychnine may be given by the mouth or by injection into the muscle we wish to develop, and care must be taken that the limb is kept warm and protected from accidents. Tubby and Jones have pointed out that a frequent cause of failure in treatment is that we are apt to leave the weakened muscles over-extended by the healthy ones; thus, if the extensors of the wrist are continually stretched no improvement can take place until this strain is taken off. If now the hand is placed on a splint in a position of hyper-extension under proper care for a long period, they will recover and, so to speak, "take up the slack." Even cases of 20 years' standing have been thus treated with success. If the extensors in such a patient show the slightest trace of voluntary power, or react on forcible flexion, they will recover. The hand may have to be kept hyper-extended for months, the muscles being massaged or percussed daily by a rubber rod. Bandages for the splint must be so arranged as to leave the extensor muscles free for this treatment, and when the patient can lift up his fingers from the splint, it is cut down to the knuckles. As he regains power in the wrist the splint can be removed altogether.

Prevention of over-stretching is as important as the cure. Hence the foot or hand must be supported by splints when any tendency of this kind exists, and the arm should be carried for a time in a sling if the biceps is weakened. The shoulder may need to be supported and the leg and foot kept in place by mechanical supports to prevent over-stretching of various muscles. In paralysis from other causes, recovery is greatly hastened if hyper-extension of the stronger muscles is carried out, and in infantile paralysis, where individual muscles are so often weakened, it is especially important. If contractions cannot be overcome by splints, tenotomy may at times be needed, and when all efforts to recover the power of a muscle have failed, transplantation of tendons or of slips from a healthy muscle may be useful.

There are few diseases in which we have greater need of patience in treatment. Though most of the improvement is gained in the first year, it often happens later on that an individual muscle or a deformity may be so treated as to give the patient a useful and sightly limb.

